# Carotid free-floating thrombus stemming from carotid web: co-occurrence of two rare causes of ischemic stroke

**DOI:** 10.1186/s12883-023-03448-4

**Published:** 2023-11-08

**Authors:** Alberto De Lorenzo, Serena Marita Lazzarin, Alessandro Bertini, Ignazio Divenuto, Simona Marcheselli, Umberto Pensato

**Affiliations:** 1 University of Milan, Milan, Italy; 2 IRCCS Humanitas Research Hospital, Via Manzoni 56, Rozzano, Milan, 20089 Italy; 3 Department of Biomedical Sciences, Humanitas University, Via Rita Levi Montalcini 4, Pieve Emanuele, Milan, 20072 Italy

**Keywords:** Ischemic stroke, Cerebrovascular disorder, Carotid artery stenting, Fibromuscular dysplasia, Young adults, Etiology

## Abstract

**Background:**

Carotid web (CaW) and carotid free-floating thrombus (CFFT) are rare yet critical causes of ischemic stroke in young adults.

**Case presentation:**

A 54-year-old woman presented with a fluctuating right sensory-motor faciobrachial syndrome. A brain MRI scan revealed multiple small recent asynchronous cortico-subcortical ischemic foci in the vascular territory of the left internal carotid artery. A CT angiography identified a CFFT in the left internal carotid artery arising from an underlying CaW. The patient was treated with excellent clinical outcomes with carotid artery stenting and dual antiplatelet therapy.

**Conclusions:**

We provide a structured pathophysiological rationale connecting CaW and CFFT and highlight pivotal therapeutic implications. Further studies are needed to investigate this relationship and guide assessment and treatment.

## Background

Etiological diagnosis of stroke in young adults is a challenging and critical aspect of neurovascular care. Indeed, patients affected by such devastating occurrences have an inherently longer time after the index event and can thus benefit most from adequate secondary prevention strategies [1]. Unfortunately, up to 40% of young adults do not reach an etiological diagnosis and are therefore left at elevated risk of stroke recurrence [2].

Carotid web (CaW) and carotid free-floating thrombus (CFFT) are well-known yet often neglected causes of ischemic stroke in young adults. CaW defines a localized carotid intimal fibromuscular dysplasia presenting as a shelf­like projection into the lumen of the proximal internal carotid artery [3, 4]. Conversely, CFFT is an elongated thrombus attached to the carotid arterial wall with circumferential blood flow at the distal aspect [5, 6].

Hereby, we describe a case of ischemic stroke due to a CFFT originating from a contiguous CaW. We illustrate the diagnostic and therapeutic implications of these two rare conditions, as well as their possible pathophysiological relationship.

## Case presentation

**Fig. 1:**
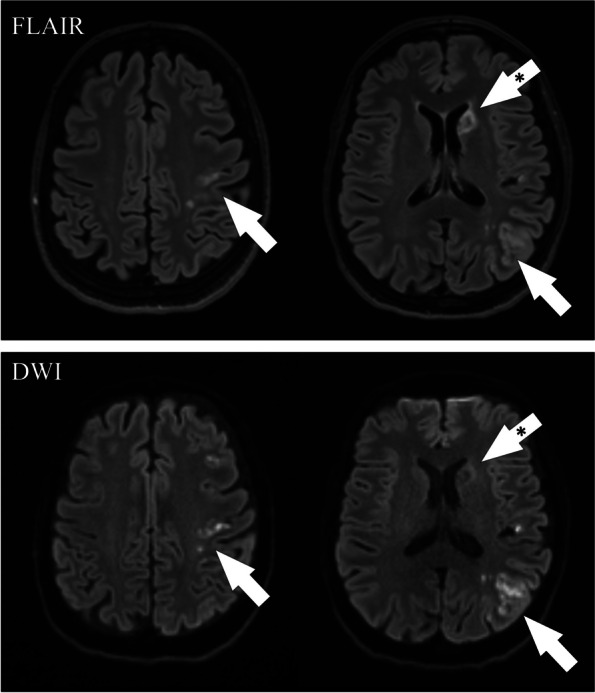
MRI on Fluid-attenuated inversion recovery (FLAIR) and Diffusion-weighted sequences showing multiple asynchronous lesions in the territory of the left middle cerebral artery (white arrows) and a subacute/chronic lesion of the head of the caudate nucleus, in the territory of the recurrent artery of Heubner (asterisk-marked arrow). All the ischemic lesions are in the left internal carotid artery territory

**Fig. 2:**
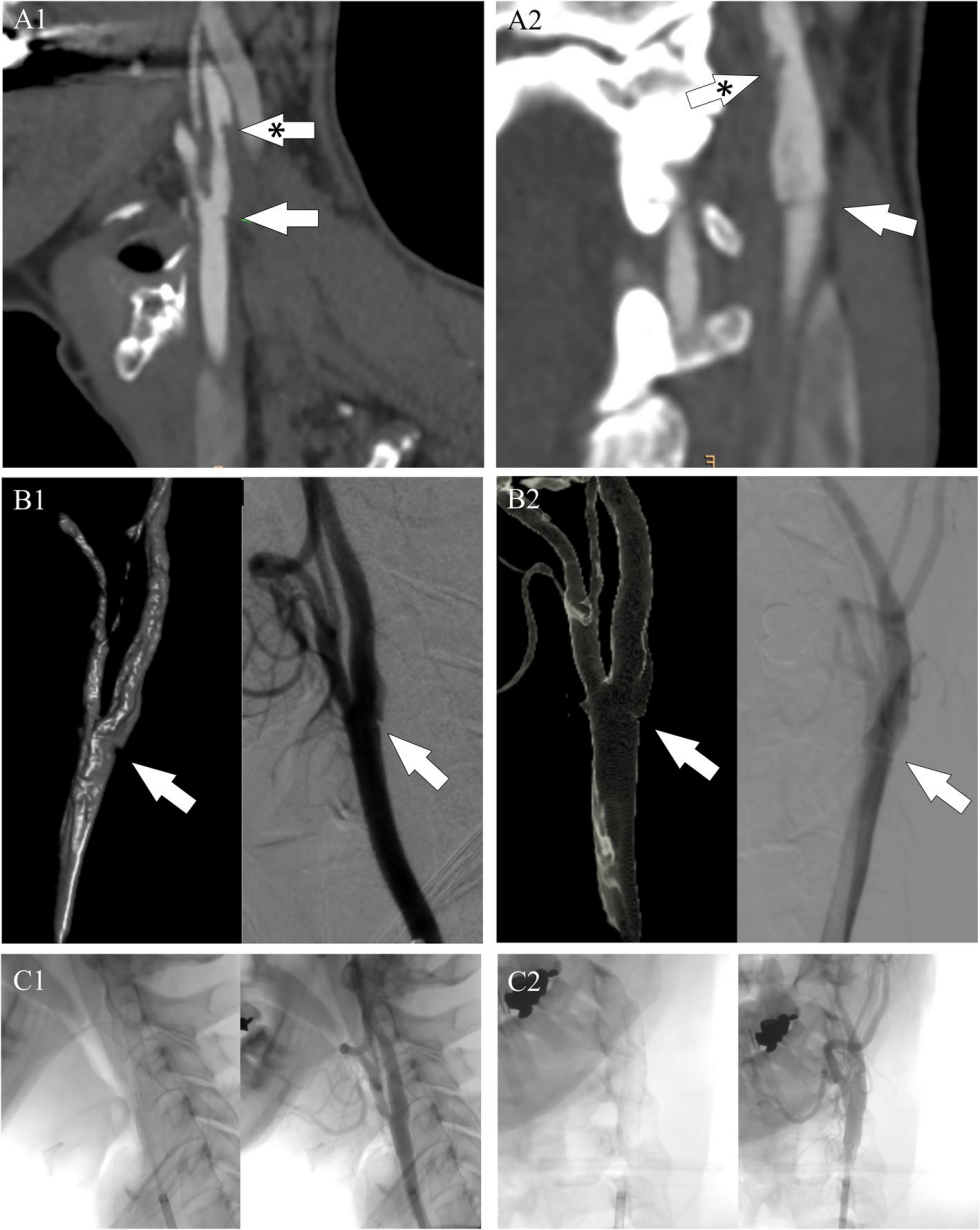
Sagittal CT (A1) and coronal 3D rotational (A2) angiography showed a linear filling defect located in the posterior internal carotid artery bulb consistent with a CaW (white arrow) and a vertically elongated hypodensity partially attached to the below carotid wall consistent with CFFT (asterisk-marked arrow). Intraoperative DSA and 3D reconstruction confirmed the presence of a CaW (arrow) on sagittal (B1) and coronal (B2) projections. Post-operative DSA showing appropriate stent placement and flow restoration on sagittal (C1) and coronal (C2) projections. CFFT: carotid free-floating thrombus. CaW: carotid web. DSA: digital subtraction angiography

**Fig. 3:**
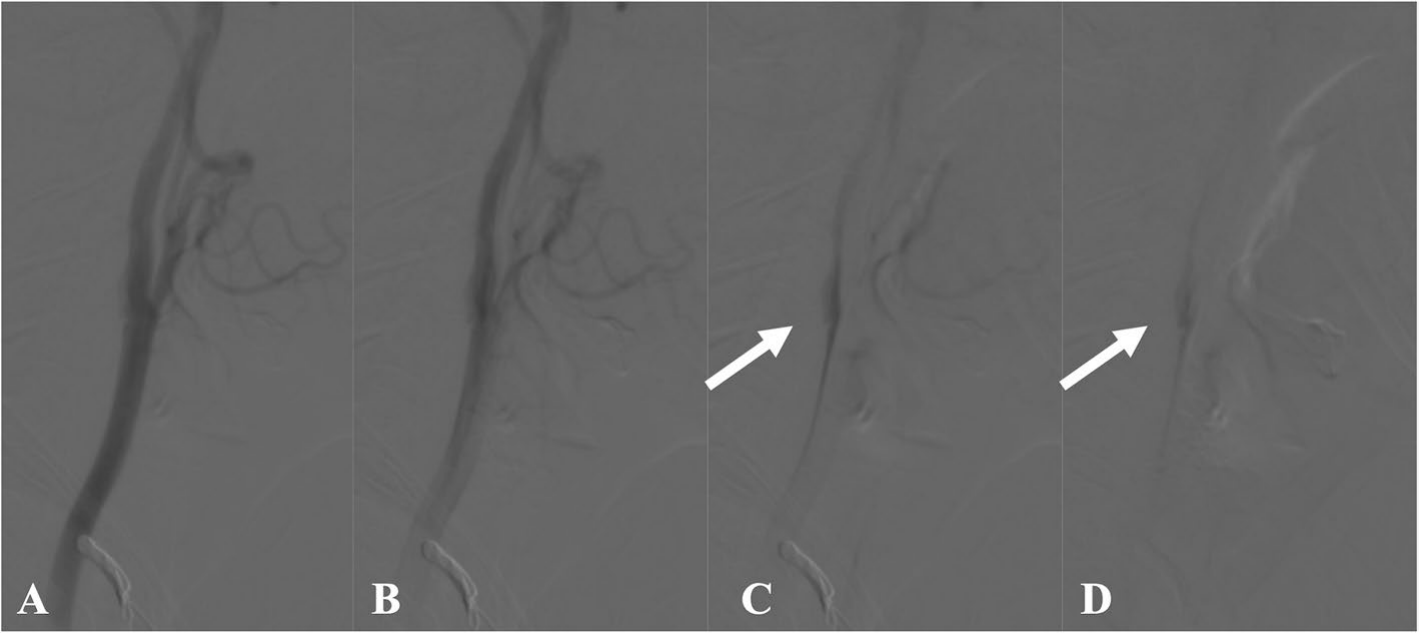
Consecutive images (**A**-**D**) acquired at two frames per second from a sagittal intraoperative DSA revealed contrast agent stagnation in the carotid web in the venous phase. DSA: digital subtraction angiography

A 54-year-old woman presented to the emergency department with a two-day history of fluctuating right sensory-motor faciobrachial syndrome. The patient was an active smoker without further major cardiovascular risk factors. Specifically, she had a normal body mass index and no medical history of hypertension, dyslipidemia, diabetes, neurological or cardiovascular events. Additionally, she was not using estrogen-containing oral contraceptives and her family history was unremarkable for cerebrovascular events. Neurological examination on admission revealed a right inferior facial palsy (NIHSS = 2). Brain CT scan and blood tests were normal. A minor stroke was suspected, therefore, the patient initiated dual antiplatelet therapy (DAPT) and was admitted to our Stroke Unit. A brain MRI scan revealed multiple small recent asynchronous cortico-subcortical ischemic foci in the left middle cerebral artery and a subacute/chronic ischemic lesion in the left head of the caudate nucleus, a territory of the recurrent artery of Heubner (Fig. 1). Continuous electrocardiography and cardiac ultrasound were unremarkable. However, carotid 2D Doppler ultrasound pointed out a possible CFFT originating from a non-stenotic hypoechoic plaque of the left internal carotid artery. CT angiography (CTA) confirmed the CFFT as a vertically elongated filling defect within the lumen of the carotid artery and detected a shelf-like projection indicative of CaW just below its origin (Fig. 2). Taking together the very high risk of stroke recurrence and the young age of the patient, a left carotid artery stenting (CAS) was performed after one week of antithrombotic treatment as secondary prevention using a 7 × 40 CGuard stent (Inspire MD, Tel Aviv, Israel) covering both the CaW and the CFFT achieving complete recanalization (Fig. 2). Intraoperative digital subtraction angiography (DSA) confirmed the presence of contrast agent stagnation during the venous phase at the CaW level (Fig. 3A-D). Conversely, the CFFT was partially resolved on DSA (Fig. 2). No CFFT or CaW was observed in the contralateral carotid artery, and no additional filling defects were detected in the extracranial and intracranial vasculature on both CTA and DSA. The neurological status ameliorated progressively and the neurological examination on discharge was unremarkable (NIHSS = 0). Notably, a comprehensive thrombophilia screening including coagulation studies, antithrombin III, protein C, protein S, antiphospholipid antibodies (lupus anticoagulant, anti-cardiolipin, anti-beta-2-glycoprotein-I), homocysteine levels and methylenetetrahydrofolate reductase gene mutation was unrevealing. DAPT was maintained for three months and later switched to a single antiplatelet regimen. At the six-month follow-up, the patient was asymptomatic and no new ischemic lesion was detected on the brain MRI.

## Discussion

We report the case of an otherwise healthy young woman who presented with multi-embolic strokes caused by a CFFT stemming from an underlying CaW. The co-occurrence of these two conditions has been reported anecdotally, and the presence of thrombus superimposition on CaW was suggested to be a contributing factor to stroke recurrence [7–10]. However, there is scarce and heterogenous evidence regarding the effectiveness of antithrombotic and/or interventional treatment as secondary prevention strategies [7–10]. CaW is a congenital variation in carotid geometry that predisposes to artery-to-artery thromboembolism by disrupting arterial laminar flow and leading to endothelial stress, blood stagnation, and downflow platelet aggregation. This rationale is supported by computational fluid dynamics analysis, which reveals an augmented recirculation zone downstream of the CaW resulting in increased wall shear stress [11]. Additionally, it is corroborated by diagnostic angiography findings, which demonstrate contrast pooling or thrombus detection downstream of the CaW [12]. CFFT, instead, is an acquired condition stemming from either systemic thrombophilia or local vascular wall dysfunction caused by atherosclerosis as well as fibromuscular dysplasia, vasculitis, dissection, and trauma [5, 6]. Hence, chronic endothelial stress withstood by the carotid section distal to the CaW arguably represented a suitable ground for CFFT formation in our patient.

Radiological differentials for CaW encompass atherosclerotic plaque with plaque rupture, carotid artery dissection, and fibromuscular dysplasia (FMD). However, the regular morphology of the filling defect, lack of calcium deposits and signs of intramural hemorrhage make atherosclerotic plaque unlikely in our patient. Furthermore, neither CTA nor DSA revealed the presence of an intimal flap, and no other stenotic segments were identified in our imaging studies, reducing the probability of carotid artery dissection and FMD.

CTA has shown superior diagnostic accuracy compared to carotid US in diagnosing CaW [13]. It is thus expected yet remarkable that the carotid Doppler ultrasound failed to identify the condition in our patient. A plausible explanation could be that the CFFT masked the contiguous congenital anomaly or that lack of knowledge of the possible co-occurrence of the two conditions limited the interpretation of the study. In line with this rationale, we argue that CaW may be a possible undiagnosed cause of CFFT when a complete radiological assessment is not undertaken, especially in young adults without traditional cardiovascular risk factors. Collectively, several radiological CTA and DSA signs helped to discriminate CaW from CFFT in our case. The CaW was located in the posterior part of the internal carotid bulb, below the origin of the CFFT. The linear filling defect was projected perpendicularly to the vessel flow direction in CaW, in contrast with CFFT. The CFFT was partially resolved at DSA, whereas the CaW was still present. Finally, contrast agent stagnation was detected in the carotid web during the DSA venous phase.

As a secondary prevention, our patient was successfully treated with CAS and transitory DAPT. Evidence of the best management is currently lacking in the literature. Indeed, secondary prevention strategies for CaW and CFFT are based on small case series and expert consensus, as no specific guidelines or clinical trials have been designed to date. Concerning CaW prevention, current strategies aim to avoid long-term complications arising from the high stroke recurrence rates. Although chronic pharmacologic management with anti-platelet agents would fall within current guidelines [14] also anticoagulant agents seem an attractive option from the pathophysiological standpoint. Nevertheless, small case series have demonstrated high recurrence rates in patients receiving solely medical treatment [3]. Accordingly, many patients are treated invasively with CAS or endarterectomy (CEA) with satisfactory results [9, 12, 15]. Although CEA was initially the primary proposed invasive treatment option, there is a growing trend towards CAS, particularly in situations involving high surgical risk or anatomical contraindications to CEA [9, 12, 15]. However, it’s worth noting that the relatively young age of many CaW patients introduces challenges, particularly regarding the necessity for long-term antiplatelet therapy [3, 4, 12]. Conversely, CFFT represents a time-sensitive finding with early and urgent management implications where both antithrombotic agents and surgical treatment have been used [5, 6]. Therapeutic implications stemming from the co-occurrence of the two conditions need thus to take into account both the urgent timeframe set by CFFT and the long-term window encompassed by CaW.

## Conclusions

We described a case of CFFT and CaW co-occurrence focusing on their possible pathophysiological connection as well as diagnostic and therapeutic implications. Meticulous evaluation of non-invasive or invasive vascular imaging tests aiming to detect an underlying CaW in unexplained CFTT should be required. Carotid artery stenting and, eventually, thrombectomy might be a safe and effective secondary prevention strategy to kill two birds with one stone unless underlying thrombophilic conditions requiring anticoagulation therapy are present. Future studies are needed to estimate the frequency and understand the underpinning biologics of the co-occurrence of these two conditions in order to inform assessment and treatment.

## Data Availability

Data will be available on appropriate request.

## Abbreviations

CaW: Carotid web
CFFT: Carotid free-floating thrombus
DAPT: Dual antiplatelet therapy
CTA: CT angiography
DSA: Digital subtraction angiography
